# Reaction Mechanism and Mechanical Property Improvement of Poly(Lactic Acid) Reactive Blending with Epoxy Resin

**DOI:** 10.3390/polym13152429

**Published:** 2021-07-23

**Authors:** Krittameth Kiatiporntipthak, Nanthicha Thajai, Thidarat Kanthiya, Pornchai Rachtanapun, Noppol Leksawasdi, Yuthana Phimolsiripol, David Rohindra, Warintorn Ruksiriwanich, Sarana Rose Sommano, Kittisak Jantanasakulwong

**Affiliations:** 1 Faculty of Agro-Industry, Chiang Mai University, Mae-Hea, Mueang, Chiang Mai 50100, Thailand; first200294@gmail.com (K.K.); thidaratkanthiya05@gmail.com (T.K.); Pornchai.r@cmu.ac.th (P.R.); noppol@hotmail.com (N.L.); yuthana.p@cmu.ac.th (Y.P.); 2 Faculty of Science, Chiang Mai University, Chiang Mai 50200, Thailand; nanthicha581@gmail.com; 3 Cluster of Agro Bio-Circular-Green Industry, Chiang Mai University, Chiang Mai 50100, Thailand; 4 Center of Excellence in Materials Science and Technology, Faculty of Science, Chiang Mai University, Chiang Mai 50200, Thailand; warintorn.ruksiri@cmu.ac.th (W.R.); sarana.s@cmu.ac.th (S.R.S.); 5 School of Biological and Chemical Sciences, Faculty of Science, Technology and Environment, The University of the South Pacific, Laucala Bay Road, Suva, Fiji; david.rohindra@usp.ac.fj; 6 Department of Pharmaceutical Sciences, Faculty of Pharmacy, Chiang Mai University, Mae-Hia, Muang, Chiang Mai 50100, Thailand; 7 Plant Bioactive Compound Laboratory (BAC), Department of Plant and Soil Sciences, Faculty of Agriculture, Chiang Mai University, Chiang Mai 50200, Thailand

**Keywords:** toughness, thermal properties, interfacial crosslink, reaction

## Abstract

Polylactic acid (PLA) was melt-blended with epoxy resin to study the effects of the reaction on the mechanical and thermal properties of the PLA. The addition of 0.5% (wt/wt) epoxy to PLA increased the maximum tensile strength of PLA (57.5 MPa) to 67 MPa, whereas the 20% epoxy improved the elongation at break to 12%, due to crosslinking caused by the epoxy reaction. The morphology of the PLA/epoxy blends showed epoxy nanoparticle dispersion in the PLA matrix that presented a smooth fracture surface with a high epoxy content. The glass transition temperature of PLA decreased with an increasing epoxy content owing to the partial miscibility between PLA and the epoxy resin. The Vicat softening temperature of the PLA was 59 °C and increased to 64.6 °C for 0.5% epoxy. NMR confirmed the reaction between the -COOH groups of PLA and the epoxy groups of the epoxy resin. This reaction, and partial miscibility of the PLA/epoxy blend, improved the interfacial crosslinking, morphology, thermal properties, and mechanical properties of the blends.

## 1. Introduction

Biodegradable polymers have attracted considerable attention in recent years owing to their environmental effects. Petroleum plastics are being replaced by renewable, eco-friendly materials. Biodegradable polymers, such as PLA [1], polybutylene succinate (PBS) [2], thermoplastic starch (TPS) [3], starch [4], polysaccharides [5], carboxymethyl bacterial cellulose [6], and pectin [7,8] have been widely studied. PLA is an eco-friendly polymer as it is synthesized using lactic acid extracted from natural sources. PLA is a typical biodegradable plastic [9] that can be used to replace commercial single-use plastics [10,11]. PLA is one of most promising candidates to replace petroleum plastic as the monomers are produced from renewable feedstock. PLA is synthesized from lactic acid using ring opening polymerization, polycondensation, and enzymatic polymerization [12]. PLA is a thermoplastic material, with high-mechanical properties, biodegradability, biocompatibility, and transparency. However, PLA has disadvantages such as brittleness, heat resistance, and barrier properties [13]. Property improvement of PLA has been investigated by several researchers [14,15,16]. Several studies have investigated the improvement of PLA brittleness and flexibility [14]. Polymer blending and co-polymerization are two methods that can be used to improve polymer mechanical properties [17,18]. Notably, PLA has been previously reported as a coating material to improve mechanical properties and water resistance of TPS [19].

Epoxy resin is a reactive polymer that contains epoxide groups in the structure, which are used to crosslink with variant functional groups such as amides and carboxylic [20]. Bisphenol A based epoxy resin is the common epoxy resin, which is derived by the reaction of thiol, alcohols, and amines with epichlorohydrin [20]. Epoxy resin is a substance that is used to improve PLA owing to its increased toughness, low shrinkage, corrosion resistance, and improved heat resistance [21,22,23,24]. Epoxy resins have epoxy groups that react with amines [25], carboxylic acids, and hydroxyl groups [26]. Epoxy interacts with some thermoplastics, such as polycarbonate [27], polyamine [28], and PLA [29]. Epoxy resin acts as a bridge to connect the polymer on both reactive sides, thus allowing the epoxy to bind different polymers [26]. In addition, chemical reactions influence the morphology, melting, and crystallization behaviors that lead to property changes [10]. Nevertheless, a high amount of epoxy resin creates a highly networked structure, resulting in a thermoset polymer. Incorporation of epoxy resin into PLA increased molecular weight, and networked and branched structures which slowed down the hydrolytic degradation of PLA by approximately 130% compared to pure PLA [30]. Epoxy resin was used as a compatibilizer to improve compatibility of the PLA/polyamide blend [31]. Reactive blending of epoxy resin and PLA increased mechanical properties, melt strength, torque, toughness, and melt viscosity, but decreased crystallinity of PLA [32,33]. These improvements were due to a reaction between -COOH end groups of PLA and epoxy groups of epoxy resin [32,33]. However, the reaction mechanism, plasticizing effect, and morphology of epoxy resin blending with PLA have not been reported.

Therefore, in this study, the effect of epoxy resin on PLA blending was investigated. PLA was melt-blended with 0–20% epoxy at 170 °C for 10 min. Mechanical properties, morphology, thermal properties, thermal stability, and the reaction mechanism were investigated.

## 2. Materials and Methods

### 2.1. Materials

Poly(lactic acid) (PLA) pellet (4032D, MW 100,000 g/mol, density 1.24 g/cc, MFI = 7 g/10 min at 210 °C, NatureWorks LLC., Minnetonka, MN, USA) was purchased from PTT Global Chemical Pub Co., Ltd., Bangkok, Thailand. Diglycidyl ether of bisphenol A epoxy resin (grade 0302, liquid state) was purchased from EASY Resin Co., Ltd., Nonthaburi, Thailand.

### 2.2. Sample Preparation

PLA was blended with epoxy resin using a two-roll mill machine (Model PII140, Pirom‒Olarn, Bangkok, Thailand) at 170 °C for 10 min, and then compressed into sheets by a hot compress at 170 °C for 10 min, followed by quenching at 10–25 °C. PLA was blended with 0.5–20% (wt/wt), a monomer of epoxy resin without a hardener to study the effects of the epoxy content. Code name and composition of the sample are shown in Table 1.

### 2.3. Tensile Properties

The tensile properties were measured following JISK-6251-7 using a tensile tester at a crosshead speed of 10 mm/min with a gauge length of 10 mm. Bone-shaped specimens of 30 × 10 × 0.2 mm (length × width × thickness) were prepared using compression molding at 170 °C for 5 min.

### 2.4. Scanning Electron Microscopy (SEM)

Morphologies of the blended samples were characterized using SEM (JSM-5910LV JEOL Co., Ltd., Tokyo, Japan) at 15 kV. The samples were broken in liquid nitrogen, followed by the coating of the fracture surface with a thin layer of gold using sputtering (108 Auto/SE sputter coater, Cressington Co., Ltd., Watford, England).

### 2.5. Differential Scanning Calorimetry (DSC)

(1) $$\%X_{c}=\left(\frac{\Delta H_{m}-\Delta H_{c}}{H_{m}^{0}}\;\right)\times 100$$

where Δ*H_m_* and Δ*H_c_* are the enthalpy of melting and cold crystallization, respectively. Δ$H_{m}^{0}$ is the melting enthalpy of 100% PLA (93.7 J/g) [34].

### 2.6. Vicat Softening Temperature

The samples with dimensions of 10 mm × 10 mm × 3 mm (width × length × thickness) were prepared using hot-compression molding at 170 °C for 5 min. The prepared samples were then tested by increasing the temperature until the flattened needle penetrated 1 mm into the surface using the ASTM D1525 standard. At least five specimens of each sample were tested.

### 2.7. Nuclear Magnetic Resonance (NMR)

The spectra were acquired using NMR (NEOTM 500 MHz, Bruker Co., Ltd., Boston, MA, USA). Samples were dissolved in a chloroform solvent (CDCL3) at 25 mg/mL before observation. Analysis of NMR intensities via different statistical models was evaluated using a custom-written Topspin 4.0.8 (Bruker BioSpin GmbH, Karlsruhe, Germany).

### 2.8. Statistical Analysis

The one-way ANOVA with the Statistical Package for the Social Sciences, SPSS Version 17 (SPSS, Armonk, NY, USA) was used to analyze the data. The differences found (*p* < 0.05) were evaluated using Duncan’s test.

## 3. Results and Discussion

### 3.1. Mechanical Properties

**Figure 1:**
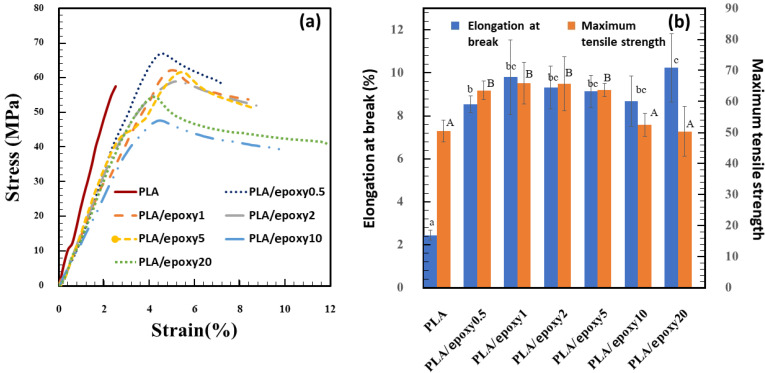
Tensile properties of PLA blend with 0–20% epoxy (**a**) Stress–strain curve, and (**b**) Maximum tensile strength and elongation at break. The different of each lowercase and each uppercase letters indicate the mean values of the elongation at break and the maximum tensile strength differ significantly (*p* < 0.05), respectively.

PLA was melt-blended with epoxy resin (0–20% *w*/*w*) to observe the effect of epoxy on the mechanical properties of the blends. The stress–strain curve, maximum tensile strength, and elongation at break are shown in Figure 1a,b. The maximum tensile strength of the neat PLA was 57.5 MPa, and the addition of 0.5% epoxy enhanced the maximum tensile strength to 66.9 MPa due to crosslinking caused by the epoxy reaction. Increasing the epoxy content extended the elongation at break of the blends, as a high crosslinking inside the PLA phase led to the formation of a network structure [35]. The Young’s modulus of PLA was 2.3 GPa, while PLA blend with epoxy 0.5, 1, 2, 5, 10, and 20% were 1.6, 1.7, 1.5, 1.6, 1.2, and 1.5 GPa, respectively. The epoxy resin reduced Young’s modulus of PLA, owing to the partial miscibility between PLA and the epoxy [24]. The high amount of epoxy acted as a plasticizer for the PLA, thereby reducing Young’s modulus and maximum tensile strength, and increasing the elongation at break, owing to its effect on the enhanced distance between the PLA molecules [29].

### 3.2. Morphology

**Figure 2:**
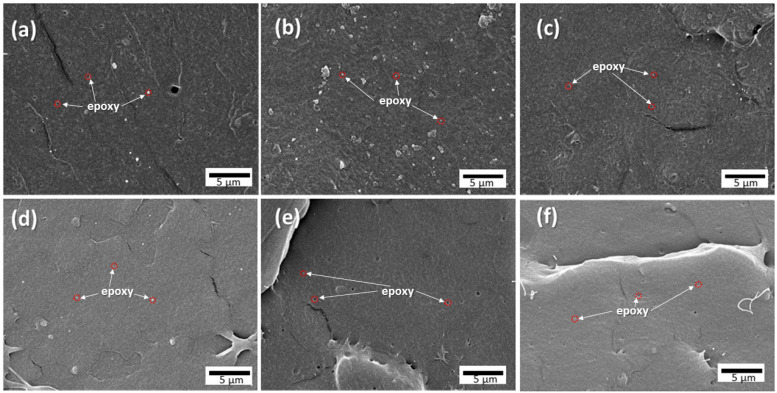
SEM fracture surface images of (**a**) PLA/epoxy0.5, (**b**) PLA/epoxy1, (**c**) PLA/Epoxy2, (**d**) PLA/epoxy5, (**e**) PLA/Epoxy10, and (**f**) PLA/Epoxy20.

Figure 2 shows the morphology of the PLA blend with 0.5–20% epoxy resin. The PLA/epoxy0.5 blend exhibited small epoxy particles (~200 nm) dispersed in the PLA matrix. Holes of the removed epoxy particles from the PLA matrix were also observed (Figure 2a). The formation of epoxy nanoparticles indicated a high compatibility between PLA and the epoxy. The PLA blend with 1–2% epoxy exhibited fine epoxy nanoparticles distributed in the PLA matrix without the removal of the epoxy particles, whereas a high epoxy content (5–20%) suggested nanoparticles that were smaller than those in the PLA/epoxy0.5 blend with a smooth fracture surface. This can be attributed to the high interfacial adhesion between PLA and the epoxy through the crosslinking interfacial reaction between PLA and the epoxy [24]. The small sizes of the epoxy particles and high interfacial adhesion resulted in the high transparency of the blend due to low light scattering [36].

### 3.3. Differential Scanning Calorimetry (DSC)

**Figure 3:**
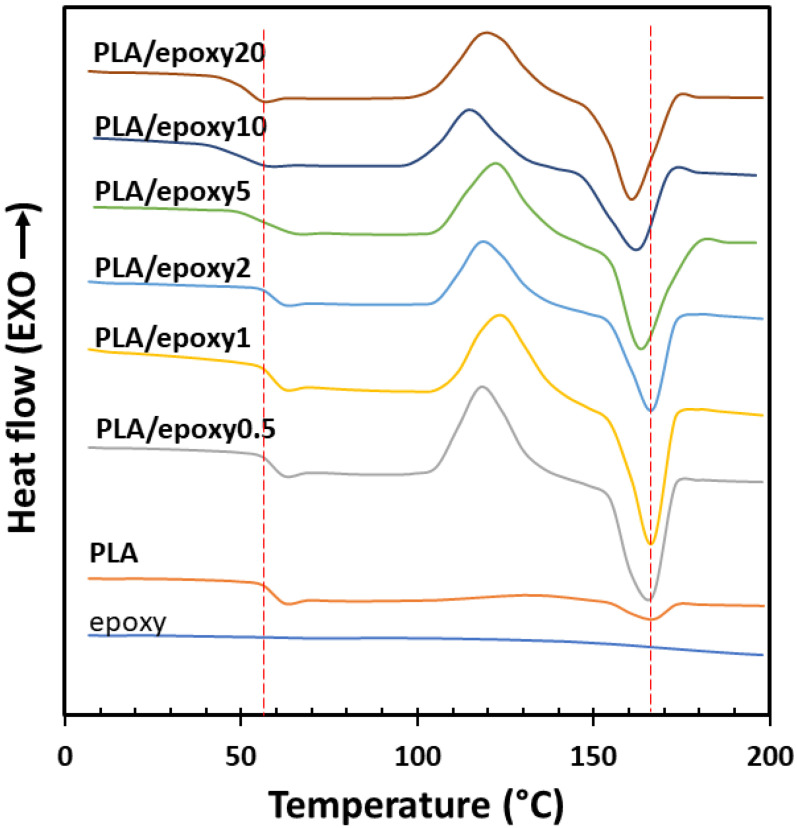
The second scan DSC curves of epoxy, PLA, and the PLA blend with 0.5–20% epoxy.

DSC curves were used to determine the effect of the epoxy resin on the thermal properties of the PLA/epoxy blends. The *T_g_* and *T_m_* were measured during the second heating scan. Figure 3 shows the DSC curves of the PLA, epoxy, and PLA blend with 0.5–20% epoxy. The *T_g_* and *T_m_* of PLA were 60 °C and 166 °C and tended to decrease with increasing epoxy content owing to small crystal sizes [37] and partial miscibility between the epoxy and PLA blends, respectively [38]. The decrease in *T_g_* indicated that the epoxy acts as a plasticizer for the PLA. Large exothermic peak of the PLA/epoxy blends indicated to recrystallization during second heating scan of DSC measurement, which presented large endothermic peak of recrystallization at 110–135 °C. This recrystallization was not observed in pure PLA.

The addition of the epoxy led to an increase in the chain length and reduced the mobility of the PLA chain [39]. The PLA crosslinked structure prevented the formation of inter- and intramolecular interactions of PLA crystallinity [40]; this resulted in the low crystallinity (0.6–3.6%) of the blends. The epoxy reaction reduced the number of PLA chain terminals in the structure and prevented the formation of nuclei as well as the growth of the crystals [29,41].

### 3.4. Vicat Softening Temperature (VST)

**Figure 4:**
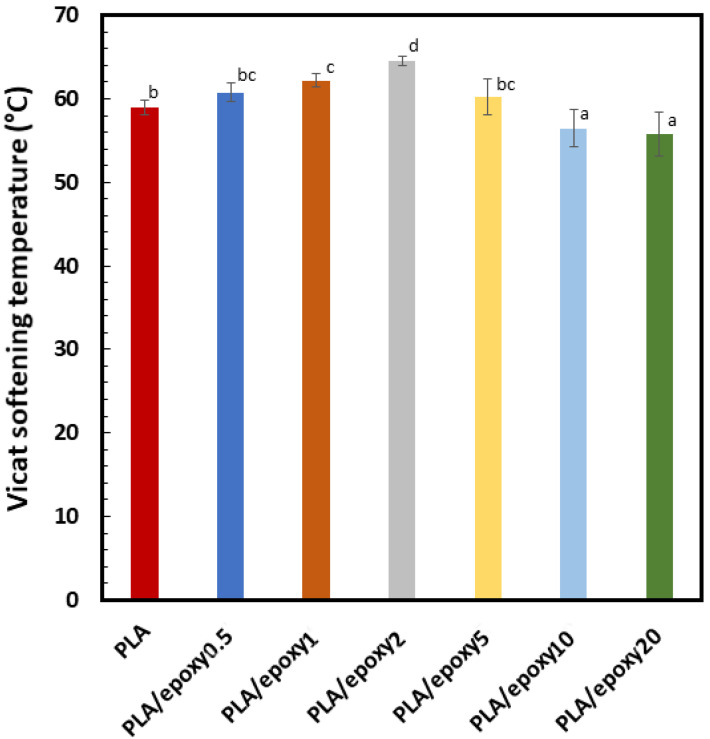
The Vicat softening temperature of PLA and the PLA blend with 0.5–20% epoxy. The mean values indicate by the different lowercase letters differ significantly (*p* < 0.05).

The VST test was used to determine the thermal stability based on the heat distortion temperature. The VST of PLA and the PLA blend with 0.5–20% epoxy resin are depicted in Figure 4. The VST of the neat PLA was 59 °C, and when 0.5–2% epoxy was added, the VST showed an increasing trend to 64.6 °C. The VST of the PLA/epoxy20 was reduced to 55.7 °C. The increased VST of the PLA/epoxy2 blend was due to the network structure of PLA, that was a result of the crosslinking reaction. Improvement of the VST due to internal crosslinking has been previously reported [42]. The decreased VST in the PLA blend with 5–20% epoxy indicated an excessive amount of epoxy and the crosslinking density inside PLA that reduced the crystal formation of the PLA. Thermal stability improvement due to a crosslinking structure and crystal formation has been previously reported [43].

### 3.5. Reaction Mechanism

**Figure 5:**
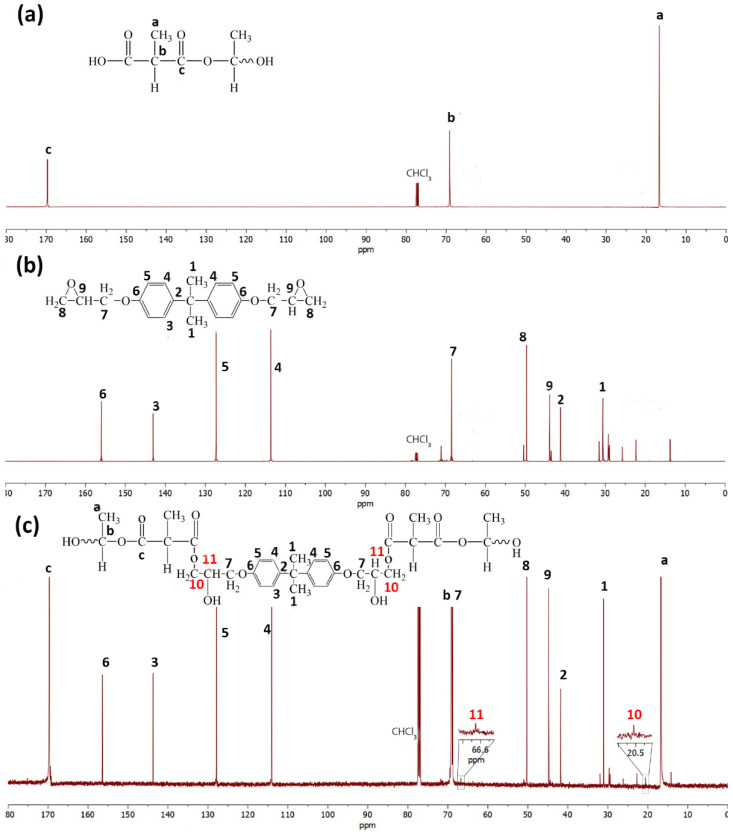
^13^C NMR spectra and structures of (**a**) neat PLA, (**b**) epoxy, and (**c**) the PLA/epoxy20 blend.

**Figure 6:**
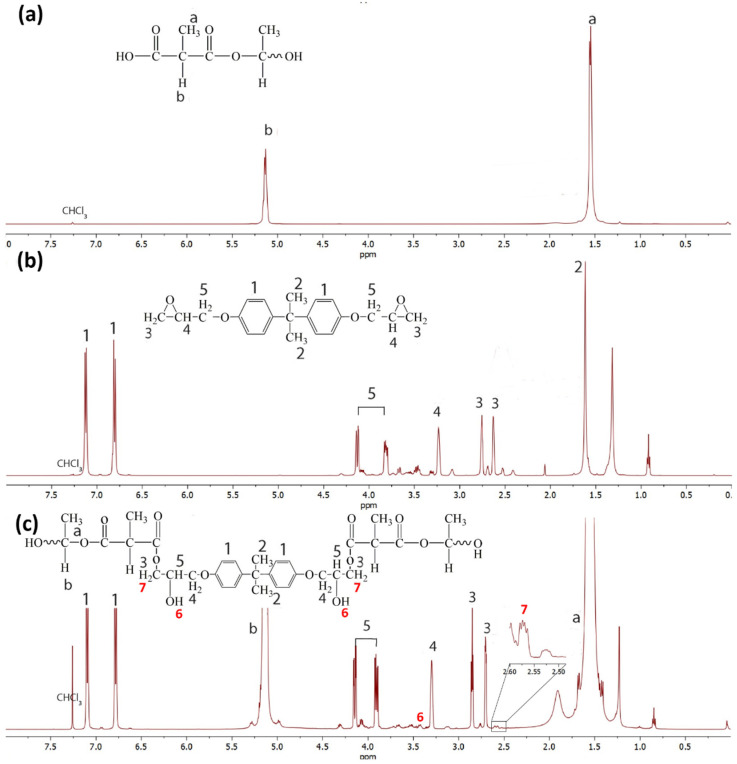
^1^H NMR spectra and structures of (**a**) neat PLA, (**b**) epoxy, and (**c**) the PLA/epoxy20 blend.

Figure 5 shows the ^13^C NMR spectra of the PLA, epoxy, and the PLA/epoxy20 blend. The epoxy showed peaks of -CH_3_ (bisphenol A) at 30.7, oxirane ring carbons at 43.9 and 49.7 ppm, and bisphenol A carbons at 41.2, 68.0, 113.6, 127.3, 143.1, and 156.0 ppm [44]. Neat PLA showed peaks at 16.7 (-CH_3_), 69.1 (methylene carbon), and 169.70 ppm (-C=O) [45]. The ^13^C NMR spectra of the PLA/epoxy blends showed the characteristic peaks of PLA and epoxy resin at the same position as that of neat epoxy and PLA. New peaks were observed at 21 and 67 ppm, corresponding to C10 and C11, respectively. Figure 6 shows the ^1^H NMR spectra of PLA, epoxy, and the PLA/epoxy20 blend. Epoxy showed peaks -CH_3_ of bisphenol A at 1.6 ppm (b), -CH_2_ of oxirane ring at 2.6 and 2.8 ppm (c), -CH of oxirane ring at 3.29 ppm (d), -CH_2_ at 3.9 and 4.29 ppm (e), and aromatic protons of bisphenol A at 6.8 and 7.1 ppm [44]. Neat PLA showed peaks at 1.6 (a,-CH_3_) and 5.1 ppm (b,-CH). The ^1^H NMR spectra of the PLA/epoxy blend showed characteristic peaks of -CH_3_ (1.6) and -CH (5.1) at the same position as that of neat PLA. Furthermore, the ^1^H epoxy peaks of bisphenol A (1.6, 6.8, and 7.1) shifted to lower positions, while the peaks of the oxirane ring (2.6, 2.8, 3.29, 3.9, and 4.29) shifted to higher positions. New peaks were observed at 2.57 and 3.40 which indicated H7 and H6, respectively. The shifting of bisphenol A and the oxirane ring, and the appearance of two new peaks, confirmed the reaction between PLA and the epoxy. This also suggested a reaction between the epoxy groups of the epoxy resin and the -COOH end groups of PLA (Figure 6c). Previous studies have reported on the reaction between epoxy groups and -COOH groups [46,47]. This reaction improved the tensile properties, toughness, morphology, and thermal properties of PLA.

## 4. Conclusions

Epoxy resin was successfully blended with PLA to improve the mechanical properties and reaction mechanism of the blends. The maximum tensile strength of PLA was improved from 57.5 MPa (neat PLA) to 66.9 MPa with 0.5% epoxy, whereas the elongation at break showed a significant increase with 20% epoxy. Morphology of the PLA/epoxy blends showed nanoparticles dispersed in the PLA matrix, while a smooth fracture surface of the 5–10% PLA/epoxy blends was observed due to the high interfacial adhesion between PLA and epoxy. The *T_m_* and *T_g_* of the PLA/epoxy blends decreased with increasing epoxy content, owing to the nucleating effect of small epoxy particle sizes, and the partial miscibility between PLA and epoxy acting as a plasticizer, respectively. The VST of PLA increased with 2% epoxy, while an excessive amount of epoxy reduced the VST due to reduced crystal formation. NMR results confirmed the reaction between the -COOH groups of PLA and the epoxy groups of the epoxy resin. This reaction improved the mechanical properties, toughness, morphology, and thermal properties of the blends, additionally resulting in high optical transparency. The PLA/epoxy blends also contained unreacted epoxy groups that could react with other reactive functional groups for reactive blending as a compatibilizer. PLA/epoxy can be applied for packaging, medical, and agriculture applications.

## Figures and Tables

**Table 1:** Code name and composition of PLA and epoxy resin blend.

Sample	PLA	Epoxy Resin
PLA	100	0
PLA/epoxy0.5	99.5	0.5
PLA/epoxy1	99	1
PLA/epoxy2	98	2
PLA/epoxy5	95	5
PLA/epoxy10	90	10
PLA/epoxy20	80	20

## Data Availability

The data presented in this study are available on request from the corresponding author.

## Abbreviations

PLA	polylactic acid
PBS	polybutylene succinate
TPS	thermoplastic starch
SEM	scanning electron microscopy
DSC	differential scanning calorimetry
*T_m_*	melting temperature
*T_g_*	glass transition temperature
NMR	nuclear magnetic resonance
CDCL3	chloroform solvent
